# Working memory load-dependent modulation of neural activity predicts response to cognitive behavioral therapy in obsessive-compulsive disorder

**DOI:** 10.1038/s41398-025-03608-9

**Published:** 2025-10-20

**Authors:** Stephan Heinzel, Christian Kaufmann, Rosa Grützmann, Björn Elsner, Benedikt Reuter, Julia Klawohn, Anja Riesel, Katharina Bey, Michael Wagner, Norbert Kathmann

**Affiliations:** 1 Department of Psychology, Humboldt-Universität zu Berlin, Rudower Chaussee 18, Berlin, Germany; 2 Institute of Psychology, Department of Educational Sciences and Psychology, TU Dortmund University, Emil-Figge Str. 50, Dortmund, Germany; 3 Department of Education and Psychology, Freie Universität Berlin, Habelschwerdter Allee 45, Berlin, Germany; 4 Department of Psychology, MSB Medical School Berlin, Rüdesheimer Str. 50, Berlin, Germany; 5 Department of Medicine, MSB Medical School Berlin, Rüdesheimer Str. 50, Berlin, Germany; 6 Department of Psychology, University of Hamburg, Von-Melle-Park 11, Hamburg, Germany; 7 Department of Psychiatry and Psychotherapy, University Hospital Bonn, Venusberg-Campus 1, Bonn, Germany; 8 Department of Old Age Psychiatry and Cognitive Disorders, University Hospital Bonn, Venusberg-Campus 1, Bonn, Germany

**Keywords:** Psychiatric disorders, Learning and memory

## Abstract

Cognitive behavioral therapy (CBT) is an effective treatment for obsessive-compulsive disorder (OCD). However, CBT does not lead to a satisfying symptom reduction in a considerable number of patients with OCD. The identification of variables that predict insufficient treatment response could improve efficient treatment selection and inform the development of specific augmentative treatments. In the current study, we tested whether prediction of treatment response can be improved by including neurobiological markers during working memory (WM) performance. Forty-four patients with a primary OCD diagnosis participated in an n-back WM task with varying WM load while functional Magnetic Resonance Imaging (fMRI) was performed. Subsequently, all patients received CBT in an outpatient clinic. WM load-dependent modulation of the blood-oxygen-level-dependent (BOLD) signal in a bilateral cluster in inferior/superior parietal lobule predicted CBT response over and above clinical and sociodemographic variables (p < 0.05). Higher modulation was associated with larger relative symptom reduction. The results of the current study indicate that the ability of the WM system to flexibly adapt to changing task demands might be a useful indicator of CBT response in OCD. Possibly, this mechanism facilitates relearning processes during exposure-based CBT. However, findings need to be replicated in larger samples.

## Introduction

Obsessive-compulsive disorder (OCD) affects 1–3% of the population [1] and is characterized by unwanted and intrusive obsessive thoughts and compulsive behaviors [2]. Cognitive behavioral therapy (CBT) is an effective evidence-based fist-line treatment for OCD [3–6]. However, about 30% of patients in CBT treatment do not show reliable clinical improvement and about 50% do not reach remission [4, 5, 7]. Thus, a substantial proportion of patients with OCD fails to benefit from CBT with negative consequences for patients and the health care system. The detection of variables that predict reduced treatment response prior to treatment can (a) support an early identification of patients with high risk for non-response and (b) improve our understanding of mechanisms that prevent treatment success [8]. With an early identification of non-respondent patients, it would be possible to offer augmented treatments to these individuals before failing with standard treatments. A better understanding of (modifiable) features that underlie unsuccessful treatments can help to identify targets for the development of additional treatment approaches tackling specific dysfunctions (e.g. targeted cognitive trainings).

Previous research on predicting treatment response in OCD has identified a set of sociodemographic and clinical variables that have shown significant results in meta-analyses and large scale studies [5, 9–11]. In brief, this research found that worse response to CBT was predicted by high OCD symptom severity, hoarding subtype, comorbid personality disorders, lower socioeconomic status, being single, and lack of treatment with psychoactive medication. However, there are relatively few consistent results across studies. Also, sociodemographic and clinical variables have not led to satisfactory prediction accuracies in most studies and information on specific targetable dysfunctions is limited. Thus, recent research has suggested that prediction of treatment response may be improved by including additional physiological and neurobiological markers [8, 12–14].

In OCD, only few prediction studies using functional Magnetic Resonance Imaging (fMRI) data have been conducted to date. Previous studies on cortical thickness [15] and resting state data [16] have demonstrated moderately better prediction performances compared to sociodemographic and clinical data. Task-based fMRI prediction studies could give more insight into specific neurocognitive dysfunctions related to CBT-nonresponse but have been sparse to date. A current meta-analysis [17] identified four studies in OCD that used task-based fMRI to predict psychotherapy response, based on fear conditioning [18], symptom provocation [19, 20], and incentive flanker [21] tasks. Taken together, a relatively intact functioning of cortical regions within the fronto-parietal network that maintain cognitive control during task performance seems to predict a better CBT response in OCD. Despite these meta-analytic findings, individual studies show partially conflicting results, e.g., concerning the role of the dorsolateral prefrontal cortex (DLPFC). While higher baseline DLPFC activity was related to a larger decrease in OCD symptoms during a cognitively demanding error-monitoring task [21], lower DLPFC activity predicted better CBT treatment response during passive viewing of emotional pictures [19]. It seems that differences in task demand may explain some of these inconsistent findings.

OCD has been related to performance decrements and alterations in fronto-striatal and fronto-parietal neural activity and connectivity during the accomplishment of working memory (WM) tasks [22–25]. As indicated by our previous report [26], OCD-related dysfunctions in WM might be best described in terms of a reduced fronto-parietal adaptability to increasing task demand, or in other words: a reduced WM load-dependent modulation of neural activity. While healthy participants usually show a stepwise increase in fronto-parietal blood-oxygen-level-dependent (BOLD) response from low to high WM load during WM task performance [26, 27], relatively increased activity at low WM load as well as decreased activity at high WM load have been found in OCD patients [22, 23, 26]. This pattern has been suggested to reflect a relevant pathological mechanism in OCD and therefore may represent a useful predictor for CBT response.

The current study aims to investigate whether neurobiological markers of WM performance predict CBT response. Based on our main findings of OCD-related alterations in BOLD response in bilateral SPL/IPL and DLPFC [26], we hypothesized that higher WM load-dependent BOLD modulation within these two core regions of the WM network [28] would predict larger percent change in OCD symptoms from pre to post CBT above clinical and sociodemographic variables. To our knowledge, this is the first study to investigate CBT response prediction with neurobiological markers during WM task performance in OCD.

## Methods

### Participants

Fifty-four patients with OCD were recruited for fMRI measurements at baseline. Two OCD patients had to be excluded from data analyses due to technical failures during fMRI scanning. Furthermore, one OCD patient showed performance at chance level (performance below 30% hit rate or above 30% false alarm rate) in the WM task, and thus, had to be excluded from data analyses as well. Seven patients with OCD did not receive CBT at the psychotherapy treatment center at Humboldt-Universität zu Berlin and no longitudinal clinical data could be obtained from these patients. Thus, the final analysis sample for treatment response prediction comprised 44 patients with OCD. All included patients had a primary diagnosis of OCD, as assessed by trained clinical psychologists at the OCD outpatient clinic at Humboldt-Universität zu Berlin, Germany, using the German version of the Structured Clinical Interview for DSM-IV TR (SCID, [29]), and had an OCD symptom severity score >12, as assessed with the Yale-Brown Obsessive Compulsive Scale (Y-BOCS, [13]). Patients were between 18 and 65 years of age, had normal or corrected-to-normal vision, reported no history of neurological diseases or brain injuries, and were eligible for fMRI scanning. Patients were excluded if they had a current or lifetime diagnosis of psychotic, bipolar, or substance use disorder, or if they took antipsychotic medication in the past four weeks or benzodiazepines in the past two weeks. The study was approved by the local Ethics Committee at Humboldt-Universität zu Berlin and conducted in accordance with the Declaration of Helsinki. Note that the current study is part of a larger project, and further results of this project have been published elsewhere [22, 26, 30–33]. Sample size was calculated to ensure adequate power to detect medium to large effect sizes. All participants gave written informed consent after receiving written and verbal information about the study and received a monetary compensation for their time. Eleven out of 44 patients (25%) discontinued treatment prematurely without the therapist’s approval (defined as non-completers), whereas 33 patients (75%) completed their treatments by a consensual termination decision of both patient and therapist (completers). All patients participated in CBT treatment including exposure and response prevention exercises as well as cognitive therapy [34]. Therapists were licensed and experienced therapists with at least three years of training in CBT and delivered CBT within regulations of the German health care system. In the current study, patients received 42 weekly therapy sessions on average (SD 18.67, range 8 – 80 sessions). Further demographic and clinical data at baseline are reported in Table 1.

**Table 1:** Socio-demographic and clinical characteristics of the whole sample (all), completers and non-completers at baseline (pre-therapy).

	All		Completers		Non-Completers	
Sex male/female	23/21		17/16		6/5	
Any comorbid personality disorder *n* (%)	10 (23%)		9 (27%)		1 (9%)	
Any psychoactive medication *n* (%)	22 (50%)		16 (49%)		6 (55%)	

	M	SD	M	SD	M	SD
Age	33.43	8.64	33.97	8.87	31.82	8.06
Number of therapy sessions	41.98	18.67	46.39	18.72	28.73	10.90
Socioeconomic status	4.80	1.89	5.12	1.75	3.82	2.04

### Data acquisition

The experimental setup, the task, information on data analysis, and results of the pre-therapy fMRI session are reported in detail in a cross-sectional investigation comparing patients with OCD to a healthy control group [26]. The 45 healthy controls were free from psychoactive medication and any current or past axis-I mental disorders, and were matched for gender, age, education, and handedness to the patient sample. In brief, all participants were scanned in a 3 Tesla Magnetom Trio Tim MR system (Siemens, Erlangen, Germany) while performing an n-back WM task with different WM load conditions (1-back, 2-back, 3-back) as well as a “0-back” condition that served as a control condition. As reported in [26], we conducted a repeated measures ANCOVA with group as the between-subject factor and WM load (1 > 0-back vs. 2 > 0-back vs. 3 > 0-back) as the within-subjects factor to identify brain regions that show OCD-related alterations in WM load-dependent BOLD response.

**Fig. 1:**
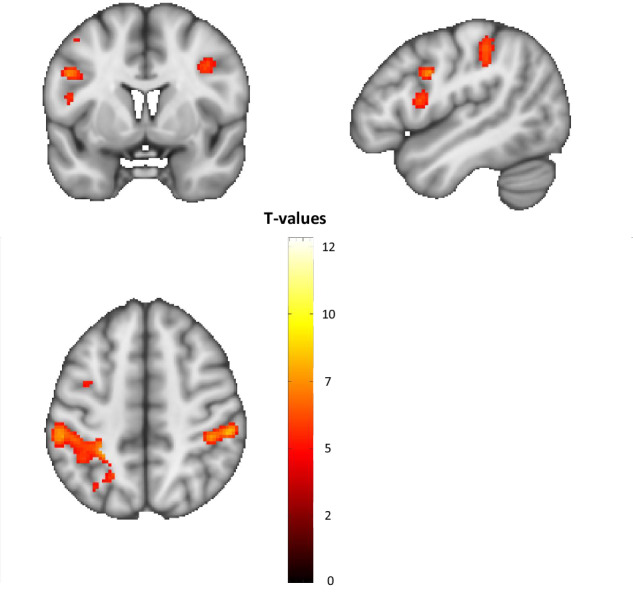
Significant clusters showing WM load-dependent alterations in BOLD response in patients with OCD (p < 0.05 FWE cluster-corrected). Figure first published in Heinzel et al. 2021 Scientific Reports under creative commons (CC BY) license. MNI coordinates (x, y, z) of peaks, cluster size: Left superior parietal lobule / inferior parietal lobule / postcentral gyrus: −24, −48, 68; 1200 voxels. Right inferior parietal lobule / intraparietal sulcus / supramarginal gyrus: 54, −32, 50; 413 voxels. Left dorsolateral prefrontal cortex: −48, 2, 32; 82 voxels. Right dorsolateral prefrontal cortex: 38, 2, 36; 55 voxels. Left premotor cortex: −42, 0, 52; 55 voxels. Left inferior frontal gyrus: −48, 8, 14; 52 voxels.

FMRI analyses revealed that patients with OCD showed a lower WM load-dependent modulation of BOLD response compared to healthy controls in six fronto-parietal clusters: Left and right superior/inferior parietal lobule (SPL/IPL), left and right dorsolateral prefrontal cortex (DLPFC), left premotor cortex, and left inferior frontal gyrus (see Fig. 1).

**Fig. 2:**
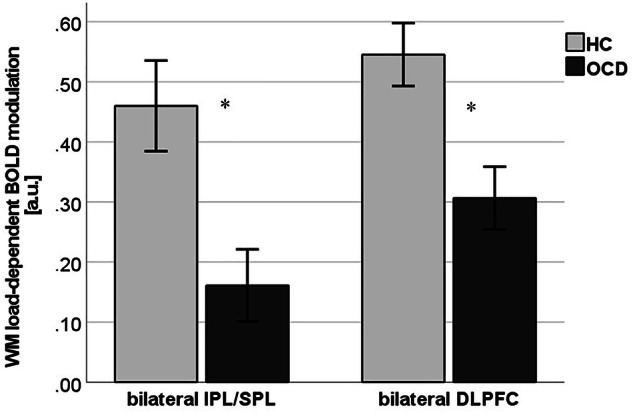
Working memory load-dependent BOLD modulation in patients with OCD and healthy controls. Working memory (WM) load-dependent BOLD modulation (3-back minus 1-back): Parameter estimates of blood-oxygen-level-dependent (BOLD) in bilateral inferior/superior parietal lobule (IPL/SPL) and dorsolateral prefrontal cortex (DLPFC) in healthy controls (HC) and patients with obsessive-compulsive disorder (OCD). *p < 0.005.

As described in the introduction, we had defined two bilateral regions of interest for subsequent analyses on CBT outcome prediction, namely bilateral SPL/IPL and bilateral DLPFC. Thus, parameter estimates (beta values) within spheres of 5 mm radius around the MNI coordinates of peak voxels of the whole-brain interaction were extracted for each WM load condition within the four anatomically defined ROIs in left and right SPL/IPL and left and right DLPFC. Following an established approach to compute a single measure for working-memory load-dependent modulation of BOLD response [27, 35], we subtracted parameter estimates during 1-back from 3-back for each of the four regions of interest. This modulation measure was used because it integrates the concepts of neural efficiency and neural capacity in WM [36]. Positive values of this measure indicate relatively low BOLD response at 1-back (high neural efficiency) and relatively high BOLD response at 3-back (high neural capacity), representing a relatively intact ability to modulate WM-related brain activity in response to changing WM load. Small positive or negative values indicate modulation impairments. In comparison to healthy controls, OCD showed a significantly reduced modulation in left SPL/IPL by −100%, in right SPL/IPL by −47%, in left DLPFC by −37%, and in right DLPFC by −46%. BOLD response in left and right SPL/IPL as well as in left and right DLPFC, respectively, were substantially correlated (r = 0.50, p = 0.001; r = 0.84, p < 0.001), and parameter estimates were averaged and the mean bilateral BOLD response in SPL/IPL and DLPFC was used for the prediction models (see Fig. 2).

### Sociodemographic and clinical measures

The following sociodemographic and clinical measures were obtained in all patients at baseline: Lifetime diagnoses of mental disorders (axis 1) as assessed with the German version of the SCID-I [29] and personality disorders (axis 2) with the SCID-II [37]. OCD symptom severity was measured with the Y-BOCS and the Obsessive-Compulsive Inventory-Revised (OCI-R, [38]), depressive symptoms with the Beck Depression-Inventory II (BDI II, [39]), and the Montgomery-Asberg Depression Rating Scale (MADRS, [40]). The primary outcome was defined as the percent change in Y-BOCS total score from pre to post therapy: Y-BOCS percent change = ((Y-BOCS post – Y-BOCS pre) / Y-BOCS pre) * 100. Thus, negative values indicate a symptom reduction from pre to post therapy measurement. Y-BOCS was assessed by trained interviewers in all patients before and after CBT. Since at least one follow-up measurement was obtained also in non-completers (interim assessments after 20 and 40 sessions), we could use the Last Observation Carried Forward method (LOCF) as an intention-to-treat measure of therapy response in all participants. All main analyses were performed in the intention to treat (ITT) sample.

### Statistical analyses

We performed two separate hierarchical linear regression analyses in the ITT sample to test if WM load-dependent modulation of BOLD-response in SPL/IPL and/or DLPFC significantly improves predicting the CBT response as defined by relative symptom change. In the first step of each of the two models, we included sociodemographic and clinical predictors as indicated by previous research [5, 9, 10]: initial symptom severity measured with Y-BOCS, socioeconomic status (composite of net income, occupational status and education with a scale from 1–7), comorbid personality disorder (none vs. at least one), and medication status (none vs. any current psychoactive medication). In the second step, we entered the BOLD modulation in bilateral SPL/IPL or DLPFC into the models.

## Results

### Symptom change

OCD symptoms as measured with the Y-BOCS (primary outcome) decreased significantly from pre to post CBT in the ITT sample (t(43) = 10.47, p < 0.001, d = −1.58). Symptoms improved by 9.89 points (absolute change) or 43.07% (relative change) on average. While Y-BOCS score post CBT was lower in completers compared to non-completers (t(42) = −2.32, p = 0.025, d = −0.81), both absolute and relative change from pre to post CBT did not differ significantly (t(42) = −1.10, p = 0.277, d = −0.38; respectively t(42) = −1.93, p = 0.061, d = −0.67). Secondary outcome measures improved significantly from pre to post CBT measurement as well in the ITT sample (OCI-R: t(43) = 8.64, p < 0.001, d = −1.32; MADRS: t(43) = 7.03, p < 0.001, d = −1.06); BDI-2: t(43) = 8.93, p < −1.36, d = −1.36). Secondary outcome measures did not differ significantly between completers and non-completers neither pre nor post CBT (all p’s > 0.23). See Table 1 for further information on baseline characteristics and comparisons and Table 2 for further information on pre to post changes in clinical symptoms.

**Table 2:** Pre- to post-therapy dimensional symptom change in primary (Y-BOCS) and secondary (OCI-R, MADRS, BDI-II) outcome measures in the whole sample (all), completers and non-completers.

	*M*_pre_ (*SD*)	*M*_post_ (*SD*)	*t*	*p*	*d*
All (N = 44)					
Y-BOCS	23.25 (5.33)	13.36 (6.77)	10.47	<0.001	−1.58
OCI-R	29.18 (11.77)	14.91 (11.19)	8.64	<0.001	−1.32
MADRS	14.18 (9.76)	5.39 (6.40)	7.03	<0.001	−1.06
BDI-II	19.84 (11.22)	8.40 (9.61)	8.93	<0.001	−1.36
Completers (N = 33)					
Y-BOCS	22.55 (5.15)	12.06 (5.60)	11.63	<0.001	−2.03
OCI-R	28.33 (11.42)	13.79 (9.91)	8.03	<0.001	−1.40
MADRS	13.45 (10.35)	5.24 (7.08)	5.41	<0.001	−0.94
BDI-II	19.42 (11.20)	7.97 (9.94)	8.49	<0.001	−1.48
Non-Completers (N = 11)					
Y-BOCS	25.36 (5.54)	17.27 (8.63)	3.04	0.013	−0.92
OCI-R	31.73 (12.97)	18.60 (14.68)	3.31	0.009	−1.05
MADRS	16.36 (7.74)	5.82 (3.92)	5.00	0.001	−1.51
BDI-II	21.09 (11.73)	9.80 (8.78)	3.23	0.010	−1.02

### Prediction of Y-BOCS percent change

**Fig. 3:**
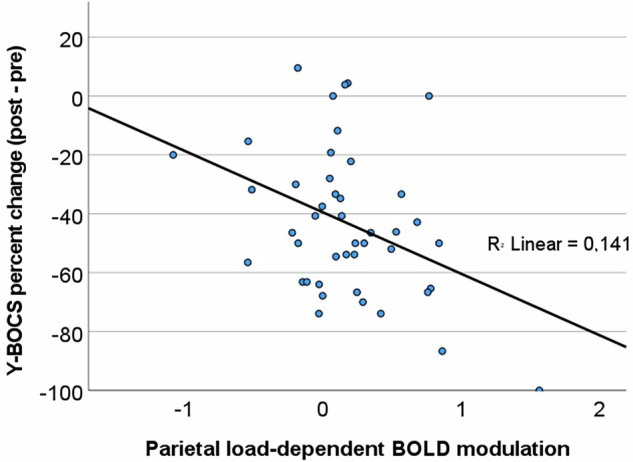
Regression from parietal BOLD modulation to symptom improvement. Regression from parietal WM load-dependent BOLD modulation to symptom improvement (percent change from pre to post CBT in Y-BOCS score).

With the exception of socioeconomic status (standardized beta = −0.32, p = 0.035), the sociodemographic and clinical variables were not found to be significant predictors of relative symptom improvement (Y-BOCS percent change), R² = 0.12, F (4, 39) change = 1.27, p = 0.299. When entering SPL/IPL BOLD modulation at the second step, the variance explained increased significantly: R² = 0.25, R² change = 0.13, F (1, 38) change = 6.97, p = 0.012. Thus, patients with a higher WM load-dependent modulation of their parietal BOLD response at baseline showed a larger relative symptom improvement post CBT. See Table 3 for all standardized beta coefficients, T-values, p-values, and effect sizes (Cohen’s d) of individual predictors. A scatter plot visualizing the relationship between parietal BOLD modulation and Y-BOCS change is shown in Fig. 3.

**Table 3:** Hierarchical linear regression models predicting Y-BOCS percent change (((Y-BOCS post CBT – pre CBT) / (Y-BOCS pre CBT)) * 100).

Regression model 1	*Beta*	*T*	*p*	*d*
Y-BOCS pre CBT	0.008	0.05	0.959	0.12
Socioeconomic status	−0.32	−2.19	0.035	−0.68
Any personality disorder	0.11	0.77	0.449	0.32
Any psychoactive medication	0.05	0.32	0.750	0.20
Bilateral IPL/SPL modulation	−0.38	−2.64	0.012	−0.82

Regression model 2	*Beta*	*T*	*p*	*d*
Y-BOCS pre CBT	0.09	0.53	0.597	0.28
Socioeconomic status	−0.32	−2.01	0.051	−0.68
Any personality disorder	0.14	0.88	0.384	0.39
Any psychoactive medication	−0.009	−0.06	0.956	−0.02
Bilateral DLPFC modulation	0.03	0.18	0.862	0.16

Entering DLPFC BOLD modulation at the second step of the second model, however, did not significantly improve prediction of Y-BOCS percent change: R² = 0.12, R² change = 0.001, F (1, 38) change = 0.03, p = 0.862. Additional explorational regression analyses including BOLD modulation in left premotor cortex or left inferior frontal gyrus or WM performance as additional predictors showed that none of these variables predicted symptom change (p’s > 0.20).

## Discussion

In the current study, 44 patients with OCD performed an n-back WM task during fMRI measurement pre CBT. We investigated if WM load-dependent fronto-parietal neural activity serves as potential biomarker for treatment response in OCD. We found that the magnitude of WM load-dependent modulation of BOLD response in bilateral SPL/IPL significantly predicted CBT response as indicated by relative decrease of OCD symptoms. Most importantly, parietal modulation significantly predicted CBT response above clinical and sociodemographic variables. Against our hypotheses, BOLD response in bilateral DLPFC did not predict CBT response in the current study.

To our knowledge, the current study is the first to investigate the utility of WM-related BOLD response as a predictor for treatment response in OCD. Our results indicate that specific neurobiological markers of OCD-related dysfunctions in WM might be useful to improve the prediction of CBT response. This extends previous research on neural underpinnings of WM deficits in OCD [22, 23, 25], indicating that an aberrant pattern of parietal WM load-dependent BOLD modulation as seen in OCD [26] may reflect a neurobiological mechanism that also affects treatment outcome.

In line with previous prediction studies in OCD [5, 10, 11], we found that a higher socioeconomic status was a significant predictor of better CBT response. Other sociodemographic and clinical variables did not predict CBT response, indicating that these variables may not consistently relate to CBT response or that contributions of these variables are small and only detectable in larger samples with higher statistical power.

As reported in more detail in our previous report [26], our finding of lateral parietal dysfunctions during WM performance may indicate deficits in selective attention, WM rehearsal, and WM capacity in OCD [28, 41]. As suggested by the meta-analysis of Harkin and Kessler [42], WM dysfunctions play a role in compulsive behavior in OCD and may be due to inefficient strategies that lead to exceeding individual WM capacity at high WM load. In line with current models, an inverse U-shaped relationship between WM load and associated BOLD responses [43–46], performance decrement and reduced BOLD response in lateral parietal and frontal regions would be expected in this case. Recent results from the ENIGMA (Enhancing Neuro Imaging Genetics through Meta-Analysis) OCD working group suggest that lateral parietal functional impairments might be related to structural changes. Boedhoe and colleagues [47] reported reduced cortical thickness in bilateral IPL as their main OCD-associated structural brain imaging finding in a sample of nearly 1500 patients with OCD.

While OCD-related alterations in DLPFC functioning during WM performance have been shown previously in several studies [25, 26, 48], WM load-dependent BOLD modulation in DLPFC did not significantly predict CBT response in the current study. This adds to the conflicting results of previous studies regarding the predictive value of DLPFC activation for CBT response in OCD [18–21], possibly reflecting task-specific differences. As the current report is the first to investigate the added value of brain response during WM for CBT response prediction, we tentatively conclude that WM-related alterations in DLPFC activity may be less sensitive to detect unsuccessful CBT outcome compared to lateral parietal dysfunctions.

Crucially, our prediction results indicate that a relatively intact parietal WM load-dependent modulation is beneficial for CBT response in OCD. Possibly, the ability of the WM system to flexibly adapt to changing task demands facilitates relearning of cognitive and emotional processing during exposure with response prevention in symptom-eliciting situations. In line with this, task-related neural flexibility has been identified as a predictor for intervention-related plasticity in cognitive training interventions [27]. At this stage, we can only speculate about the role of WM during CBT in OCD, but experimental research on WM and emotion regulation in depression suggests that intact selective attention and WM capacity functions improve the success in down-regulating negative emotions [49–51]. As recently shown, this ability might be helpful to achieve reliable improvements during treatments with in vivo exposure [52]. It has been stated that a certain degree of functioning of the neural circuitry supporting cognitive control and emotion regulation capacities may be a “gateway” to receiving benefit from psychotherapy [53]. Thus, a certain degree of functioning of these circuits may be required to achieve an optimal outcome of CBT [54].

### Limitations and future perspectives

There are some limitations that need to be considered when interpreting the results of this study. Mainly, the sample size is relatively small, thus preventing us to apply more complex statistical prediction analyses. Results need to be replicated in larger samples that would allow a cross-validation of predictors and machine-learning approaches, for example. Also, no waiting list control group was included in the current study. Thus, part of the reported symptom change could have been caused by other factors than the CBT intervention (e.g. spontaneous remission, expectancy, unspecific intervention effects). Furthermore, it needs to be noted that even though WM-related neurobiological markers showed large and significant effects, the magnitude of explained variance in symptom reduction was relatively small. While our results are highly relevant for research in the field of translational psychiatry, the integration of additional data levels (e.g. sensor data, ecological momentary assessments, genetic data) may be required [55] to achieve acceptable prediction accuracies on the single case level that ensure clinical utility. Finally, it would be relevant to test whether the findings are specific to OCD by also including patients with other diagnoses such as depressive disorders and anxiety disorders in future prospective prediction studies. If the main findings of the current study prove to be reliable and valid by replication studies, development and testing of specific cognitive trainings that counteract the identified neural dysregulations [27, 56] could be a promising next step to improve CBT response in OCD.

## Conclusion

In the current study, it was found that WM load-dependent modulation of BOLD response in inferior/superior parietal lobule predicted CBT response, over and above sociodemographic and clinical variables, such that lower modulation was associated with smaller relative symptom reduction. This finding is of high relevance because it sheds light on a potentially critical pathological mechanism in a subgroup of patients with OCD showing diminished response to CBT treatment.

## Data Availability

Analysis scripts are available on OSF at https://osf.io/rkx3b/ and data upon request from the authors.
